# Posterior Urethra Rupture: Contrast-Enhanced Computed Tomography Scan and Urethrocystography Demonstrations

**DOI:** 10.1155/2012/109589

**Published:** 2012-03-26

**Authors:** Wojciech Marks, S. Dawid, J. Lasek, Z. Witkowski, K. Gołąbek-Dropiewska, M. Stasiak

**Affiliations:** Department of Trauma Surgery, Medical University of Gdańsk, 80-211 Gdańsk, Poland

## Abstract

In the follow-up study of patients with pelvic fractures, rupture of the posterior urethra is registered in 3–25% of cases (Koraitim et al., 1996). The diagnostic gold standard for the assessment of hemodynamically stable trauma patients is contrast-enhanced CT scan, especially helical CT. Nevertheless, simultaneous suprapubic cystography and ascending urethrograms (the so-called up-and-downogram) are the investigation of choice in assessing the site, severity, and length of urethral injuries. (Carlin and Resnick, 1995) This paper discusses the evaluation and diagnosis of urethral injury in multiple-trauma patient.

## 1. Introduction

Urethral lesions may be caused by blunt (90%) or penetrating objects in the course of accidents, or can be iatrogenic resulting from invasive measures [1, 2]. Unlike the bulbar urethral injury, nearly all ruptures of the membranous part of the urethra are concomitant with a pelvic fracture [3]. Diagnostic clarification of the exact nature of urethral injuries requires high-quality imaging studies by specialists in this field. Simultaneous suprapubic cystography and retrograde urethrography (the up-and-downogram) are the choice diagnostic in evaluation of the site, severity, and length of the injury. However, the retrograde urethrocystography is also accepted as a gold standard in the diagnosis of urethral injuries [3–6]. Computed tomography imaging however plays a key role in management of multiple-trauma patients not only in the diagnosis of urinary tract injuries but also primary identification of other internal organs damage [7]. This paper presents in the evaluation in posterior urethra rupture in multiple-trauma patient.

## 2. Case Report

A 35-year-old patient was admitted to hospital because of multiple injuries he sustained in car crash. He was unconscious, GCS 5 points. Clinical examination suggested severe intracranial trauma, lungs contusion, and right crus fracture. Also one failed urinary catheterization was attempted and blood appeared in catheter during this procedure. A CT scan was performed. Investigation detected fracture of left pubic bone and the presence of the catheter balloon inside the urethra (beside intracranial and chest injury). After second catheterization contrast has been admitted through urine catheter, but the balloon still has been placed in urethra; and there was no contrast inside urine bladder visible. That suggested urethra detachment. In this situation cystostomy has been performed.

**Figure 1:**
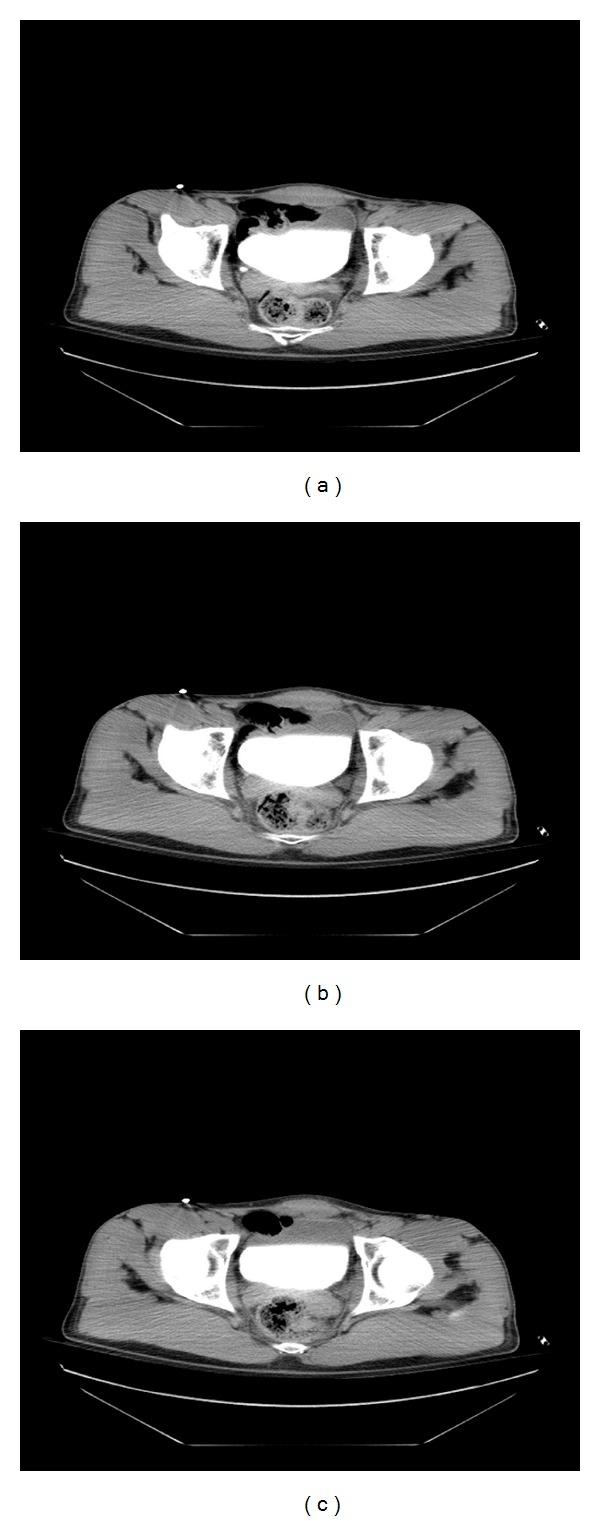
Contrast-enhanced CT scan (Siemiens). Contrast fills bladder without outflow. Urethra has not been revealed on these scans.

**Figure 2:**
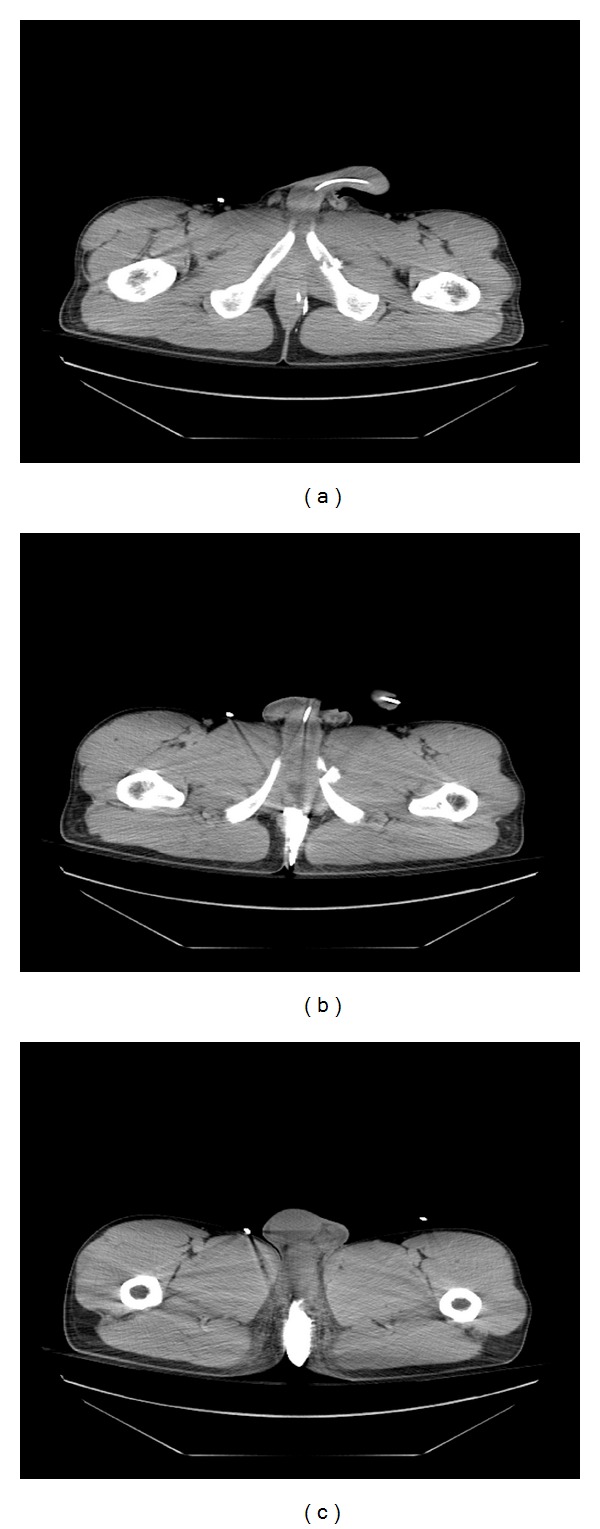
CT Urethrocystography (Siemiens). Contrast fills urethra without filling bladder. Left pubic bone has been detected in (a) and (b).

In the next period 2 complementary diagnostic methods were performed. Contrast-enhanced CT scan (Siemiens)-investigation demonstrated that contrast fills bladder without outflow. Urethra has not been revealed on these scans (Figures 1(a)–1(c)). And CT Urethrocystography (Siemiens) examination demonstrates that contrast fills urethra without filling bladder (Figures 2(a)–2(c)). After careful analysis of patient general condition and all injuries; preservative treatment has been introduced.

**Figure 3:**
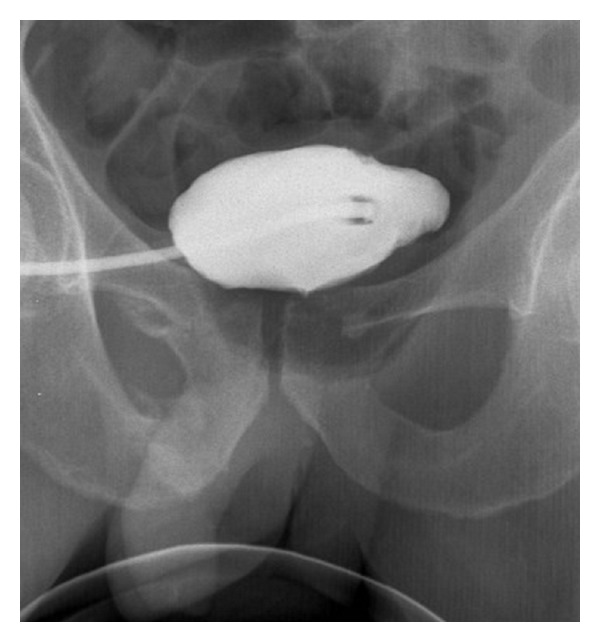
Control urethrocystography (2 weeks later). Examination performed with the patient supine. Contrast which is given through Pezzer catheter fills bladder without outflow. Urethra also has not been revealed on these scans.

Control urethrocystography (2 weeks later) showed that contrast administrated through Pezzer catheter fills bladder without outflow (Figure 3). Urethra also has not been revealed on these scans. 

The patient stayed in the hospital for 3 months because of infectious complications concerning both lungs and urine tract. The patient was consulted by urologists; and the decision of urethra continuity restoration was made after finishing treatment of other injuries and complications and proper preparation. Until this moment cystostomy is maintained.

## 3. Discussion

 Multiple trauma is the growing problem nowadays as a result of technical progress, higher speeds, extreme sports spreading, and usage of alcohol and other psychoactive substances. Helical CT remains the gold standard diagnostic tool in hemodynamically stable trauma patients, and immediate surgical exploration should be performed in instable cases. The mechanism of injury in car accidents or falls from the height predisposes to pelvis fractures and internal organs damage. CT imaging plays also a key role in diagnostic tool of urinary tract injuries. The CT findings of alteration or obstruction of the urogenital diaphragm fat plane, (hematoma) of the ischiocavernosus muscle, the bulbocavernosus muscle, obturator internus muscle; and distortion or obscuration of the prostatic contour are more common in patients with pelvic fractures associated with urethral injuries than in patients with uncomplicated pelvic fractures [2–7].

Retrograde urethrography is complementary diagnostic method, which should be used when there is a strong suspicion of urinary tract trauma. In cases where pelvic fracture is accompanied with either blood at the external urethral meatus or other signs of probable urethral injury, a retrograde urethrogram should precede the insertion of a urethral catheter. When no changes are revealed, urethral catheter may be placed into the bladder; and a retrograde cystogram is performed in order to reveal eventual associated bladder injury [3, 5–7]. In our patient (as in many others) nevertheless there were no external symptoms of urethral injury at admission. So failed urine catheterisation and the occurrence of blood in the catheter led to suspicion of urethra damage that was confirmed in CT and retrograde urethrography.

In retrograde urethrography urethral injury is demonstrated by extravasation of contrast medium. A complete urethral tear appears as contrast extravasation without filling of the bladder; whereas in an incomplete tear contrast fills partially the proximal urethra and bladder [6, 7].

The classification of posterior urethral injuries is based on the degree of changes demonstrated by the retrograde urethrogram (Sagalowsky and Peters, 1998). A type I injury is the least severe and includes urethral stretching and elongation without rupture. The type II urethral injury is a consequence either of partial or complete rupture of the prostatomembranous urethra with the extravasation on the retrograde urethrogram restricted below the urogenital diaphragm—‘‘pie-in the sky” floating bladder and prostate. Type III urethral injury is the most severe in which urethral tear is combined with a rupture of the urogenital diaphragm and bulbous urethra [5]. In our patient type II urethral injury has been recognised.

In the treatment of posterior urethral injuries two strategies are taken into account—primary and delayed reconstruction. Nevertheless both have their pros and cons [3, 6]. 

The primary urethra realignment and its stabilisation on the catheter reduce the degree of stricture formation and also avoid the lengthy period of suprapubic catheterization. Nevertheless when there is no indication for immediate surgery (other organs damage) this should be considered only in stable patients with minor injuries in other areas that are capable of surviving a prolonged anaesthesia and placement in the lithotomic position [5].

 In severely injured multiple trauma patients the delayed urethra reconstruction is the treatment of choice. The two methods are to consider: the delayed primary reconstruction and secondary restoration of the urethra. The former is performed in about 7–10 days after trauma. This approach usually permits stabilization of the patients' state as well as performing surgical repair before the development of fibrosis between and nearby the distracted urethral ends. Unfortunately in many patients (as in our case) after severe polytrauma their condition and early complications of other injuries do not allow long-standing anaesthesia and intraoperative blood loss [3].

In those cases secondary repair should be introduced. The prolonged suprapubic cystostomy maintenance is needed in this situation. The operation is performed 2-3 months after injury when the fibrotic tissue is stabilized. This approach is established as a safe strategy and also effective at decreasing the rates of all major complications [3, 5].

## 4. Conclusions

The current paper presents an appropriate approach to trauma patients with posterior urethra involvement. Helical CT remains the gold standard diagnostic tool in hemodynamically stable trauma patients, and immediate surgical exploration should be carried out in instable cases. Retrograde urethrography is complementary diagnostic tool, which should be used in certain urinary tract trauma.

**Learning Points**

- Simultaneous suprapubic cystography and ascending urethrograms (the so-called “up-and-downogram”) are the investigation of choice in assessing the site, severity, and length of urethral injuries.
- The diagnostic gold standard for the assessment of hemodynamically stable patients is Contrast-enhanced CT scan, especially helical CT.
- Diagnostic clarification of the exact nature of urethral injuries requires high-quality imaging studies by specialists in this field.
